# Editorial: Individualized Psychotherapy Treatment of Young People With Mental Disorders

**DOI:** 10.3389/fpsyg.2022.838296

**Published:** 2022-03-23

**Authors:** Giada Pietrabissa, Stefanie Julia Schmidt, Henriette Loeffler-Stastka, Randi Ulberg

**Affiliations:** ^1^Department of Psychology, Catholic University of Milan, Milan, Italy; ^2^IRCCS Istituto Auxologico Italiano, Milan, Italy; ^3^Department of Clinical Psychology and Psychotherapy, University of Bern, Bern, Switzerland; ^4^Department of Psychoanalysis and Psychotherapy, Medical University Vienna, Vienna, Austria; ^5^Faculty of Medicine, Institute of Clinical Medicine, University of Oslo, Oslo, Norway

**Keywords:** psychotherapy research, youth, mental health, engagement, treatment outcomes, drop out

Applied practice-oriented psychotherapy research is of great significance in youth mental health, as research in this specific age group lacks dismantling studies for prosperous or hindering factors concerning engagement into change processes and efficacy. The advancements of the work consist of conceptual considerations for the treatment planning, assessment of several also subjectively relevant factors, the importance of the accessibility and preparedness of the (social) environment, as well as intrapsychic resistance or resilience factors. Qualitative studies and research designs of all evidence levels provide insight into the facilitation of treatment for young people.

This Research Topic provides an overview of studies on psychotherapeutic treatment for young people and employs different research methods for an in-depth investigation of predictors for successful outcomes, barriers and facilitators, and factors enabling engagement of young people in psychotherapy. Specific recommendations on how to effectively measure mental phenomena and address psychological difficulties in this particular age group are also investigated and provided. Indeed, articles included in this Research Topic focus on testing how to assess emotional factors, besides configuring future contributions to fill research gaps in this area that also consider mediators and theories of change in psychotherapy.

Overall, psychotherapy has been found effective for the treatment of mental health problems in youth.

The systematic review of studies proposed by Midgley et al., for instance, provides a narrative synthesis of the evidence for the effectiveness of psychodynamic psychotherapy in treating a wide range of mental health difficulties in children and adolescents. Findings from this study also highlight that this approach may be especially effective for internalizing disorders such as depression and anxiety, for treating emerging personality disorders or children who have experienced adversities.

In the contribution by Gergov et al. psychological symptoms and distress also decreased significantly during the course of the treatment (including psychodynamic psychotherapy, cognitive, crisis- and trauma-focused therapy, family therapy, art, and occupational therapies), with better results occurring within the first 6 months from treatment initiation. Intensive psychotherapy offered for a shorter period was the strongest predictor of good outcomes among the respondents, while adolescents with a higher level of externalizing problems or lower level of expectations for their active role in the treatment had a higher risk of dropping out.

While findings from the above studies rely solely on quantitative data, an empirically informed conceptual psychotherapy research method (Leuzinger-Bohleber and Fischmann, 2006) was used in the contribution by Messina et al. to illustrate how technical aspects of intensive transactional analysis psychotherapy (ITAP) and the therapist's attitude might lead to different therapeutic processes and outcomes through two clinical cases conducted on young adults.

This strategy is very important nowadays when it comes to establishing an individualized approach in treatment that should gain a solid scientific stance compared to adding to precision medicine the importance of subjective meanings. In this longitudinal study, both quantitative and qualitative data are used to obtain a comprehensive picture of the effectiveness of ITAP—and the patients' ego strength is seen as an important variable to be considered for an effective treatment process.

Still, despite exploring treatment options for mental health problems in youth is of primary importance, clinicians and researchers also need to be aware that the majority of young people fulfilling the criteria for psychological disorders do not ask for help/receive treatment and a large proportion (28% up to 75%) of the treatments in youth mental health care results in premature termination (Block and Greeno, 2011).

Indeed, the dropout phenomenon and the importance of engaging youth in psychotherapy are also examined and discussed by the studies included in this Research Topic. Contributions try to beg the question of what differentiates helpful from unhelpful treatment processes from the perspective of young clients (Stige, Barca, et al.) and explore potential therapist strategies and behaviors to engage adolescent clients who come to therapy at the initiative of others (Stige, Eik, et al.).

Fredum et al. focus on the therapy short-term psychodynamic psychotherapy (STPP) process and the interaction between adolescents with major depressive symptoms and the therapists using a quantitative method and concluded over the multidimensional nature of premature drop-out in youth.

Still, additional requirements for specific treatment strategies is the assessment of first-person perspective and subjective parameter providing insight into the question of how to involve young patients in psychotherapy. In this regard, findings from Stige, Barca, et al. contribution highlight the key role played by the therapist in engaging youth in psychotherapy by ensuring an individualized treatment that meets the needs of adolescent clients, but also further shed light on the importance for adolescents to feel active participants in therapy. Moreover, results from this study stress the extent to which service organizations should allow sufficient flexibility for therapists.

Public/patient involvement plans and research designs are, therefore, necessary and should be facilitated, fostered, and strengthened to grasp differentiated effects of subjective meaning thus bringing all the goods to the client (Sales et al.).

Accordingly, a series of focus groups were conducted in the study by Stige, Eik, et al. to explore the way therapists manage to engage adolescents in therapy. Results led to the emerging of four main themes: counteracting initial obstacles for client engagement, sharing definitional power, practicing transparency, and tailoring as ideal. However, once again, system requirements, and services organization were found to obstruct and influence these processes in several ways, pointing to the significance of exploring the interplay between system organization and therapeutic practice more thoroughly.

As part of this Research Topic, another interesting work on how to include patients' perspectives in the study of the mind is provided by Löffler-Stastka et al. It offers practical suggestions for the design of research able to incorporate the first-person account—a major step toward a better understanding and treatment of mental problems. It is also aimed to review qualified phenomenological methods for the acquisition and interpretation of experiential data in patients with depression.

Environmental and systemic factors and the proper engagement of young patients in therapy are all factors that contribute to successful treatment outcomes. Also, as shown in the study by Edbrooke-Childs et al., young people accessing mental health services whose symptoms meaningfully improve seem more likely to mutually agree to end treatment.

In order to develop differentiated psychotherapy research, fill research gaps and facilitate further precise treatment options, reliable measures of outcomes and/processes also need to be developed and tested. In the present Research Topic, the construct validity of the Mentalization Scale (Richter et al.). Validation of the multicultural quality of life index in Norwegian pediatric mental health settings are offered (Mundal et al.).

Mediators of treatment outcome and theories of change in psychotherapy for young people suffering from mental problem, including personality disorders (Volkert et al.), anxiety and depression (Conejo-Cerón et al.) also need to be investigated and summarized to gather and display the common implicit knowledge of psychotherapy research done till now. These studies also help to shed a light onto gaps concerning the efficacy of psychotherapeutic factors, patient or therapist variables, and point at prosperous but also hindering factors for treatment success.

## Author Contributions

GP and HL-S produced the first draft the editorial. SS and RU reviewed the article. All authors contributed to the article and approved the submitted version.

## Conflict of Interest

The authors declare that the research was conducted in the absence of any commercial or financial relationships that could be construed as a potential conflict of interest.

## Publisher's Note

All claims expressed in this article are solely those of the authors and do not necessarily represent those of their affiliated organizations, or those of the publisher, the editors and the reviewers. Any product that may be evaluated in this article, or claim that may be made by its manufacturer, is not guaranteed or endorsed by the publisher.
